# Childhood cancer mortality in Japan, 1980–2013

**DOI:** 10.1186/s12885-015-1472-x

**Published:** 2015-06-01

**Authors:** Limin Yang, Junichiro Fujimoto

**Affiliations:** 1 Division of Allergy, Department of Medical Subspecialties, Medical Support Center for Japan Environment and Children’s Study (JECS), National Center for Child Health and Development, 2-10-1 Okura, Setagaya-ku, Tokyo 157-8535 Japan; 2 Epidemiology and Clinical Research Center for Children’s Cancer, National Center for Child Health and Development, 2-10-1 Okura, Setagaya-ku, Tokyo 157-8535 Japan

**Keywords:** Cancer, Children, Mortality, Trend

## Abstract

**Background:**

This study aimed to provide an updated analysis of childhood cancer mortality rates and long-term trends to 2013 to describe the current level of deaths and identify changes in recent decades.

**Methods:**

Data on number of deaths from cancer in children aged under 15 years were derived from Vital Statistics in Japan and the World Health Organization (WHO) mortality database for comparison countries. Trends in mortality were examined by fitting a joinpoint regression model.

**Results:**

For all cancers combined, the mortality rate during 2010–2013 was 19.9 per 1,000,000 population for boys and 17.5 for girls in Japan. Mortality from all cancers combined decreased significantly from 1980 to 2003 for boys and from 1980 to 2001 for girls. Afterwards, the rates remained stable for both sexes. Mortality from leukemia declined over the entire study period by 4.6 % per year (*p* <0.05) in boys and 4.3 % per year (*p* <0.05) in girls. For central nervous system (CNS) tumors, a slight increase in mortality was observed for both sexes, with a statistically significant annual percent change (APC) of 0.5 % (*p* <0.05) for boys and 0.6 % (*p* <0.05) for girls.

**Conclusions:**

We provided updated information on recent trends of childhood cancer death. The establishment of a nationwide, childhood cancer registry is required in Japan. Moreover, trends in cancer mortality should be monitored continuously.

## Background

Compared with cancers that occur in adults, childhood cancers are rare, comprising only 0.2 % of all cancers in Japan in recent years [1]. However, cancer is the commonest cause of death among Japanese children aged 1–14 years, accounting for 17 % of deaths, according to the Vital Statistics of 2013 [2]. About 2,300 children under 20 years of age were diagnosed with cancer in 2011, according to a recent report from the Medical Aid Program for Chronic Pediatric Diseases of Specified Categories, a research project conducted by the Ministry of Health, Labor and Welfare, Japan [3].

Although the above Medical Aid Program reports the number of new cancer patients, data quality is a problem [4]. The Japan Society of Pediatric Hematology/Oncology has also made great efforts to collect data on the incidence of childhood cancer, but its registry includes only some new patients [4]. In fact, Japan has no nationwide, childhood cancer registry and thus no accurate estimates of cancer incidence in Japanese children. Therefore, evaluation of childhood cancer mortality trends is especially important.

Following our previous report on childhood cancer mortality in Japan between 1970 and 2006 [5], this article provides an updated analysis of mortality rates and long-term trends up to 2013.

## Methods

Data on all deaths in Japan from cancer by sex at ages 0–4, 5–9 and 10–14 years during 1980–2013 were obtained from Vital Statistics compiled by the Japanese Ministry of Health, Labour and Welfare [2]. Death registration is required by “Family Registration Law” in Japan [2]. The registration of vital events was believed to be complete [6, 7]. Population data by sex and age were available from a Japan national population census, which is conducted almost every 5 years, and from intercensal estimates. We calculated age-standardized mortality rates by the direct method using age-specific mortality rates for 5-year age intervals and weights based on the age distribution of the standard world population (WHO 2000–2025) [8]. We also calculated average age-adjusted rates, which included age-adjusted rates for six 5-year periods from 1980–1984 through 2005–2009 and the 4-year period of 2010–2013.

We analyzed the following malignant tumors: all cancers combined (International Classification of Diseases (ICD)-9: 140–208; ICD-10: C00–97), leukemia (ICD-9: 204–208; ICD10: C91–C95), lymphomas (ICD-9: 200–202; ICD-10: C81–85, C96), central nervous system (CNS) tumors (ICD-9: 191–192; ICD–10: C70–C72), malignant kidney tumors (ICD-9: 189; ICD-10: C64–C66, C68), and malignant bone tumors (ICD-9: 170; ICD-10: C40–C41).

Trends in mortality were examined by fitting a joinpoint regression model [9]. A maximum of four joinpoints were allowed, and a minimum of six observations were required between two joinpoints (including any joinpoint that falls on an observation) . Permutation tests were used to select the number of joinpoints. We calculated the average annual percent change (AAPC) from 2000 to 2010 and during the last five observations.

For comparison with other countries, we analyzed 1980–2010 mortality data for the United States (US), the United Kingdom (UK), Italy, and France. Data on deaths and population were obtained from the World Health Organization (WHO) mortality database [10]. Because death is rare from childhood cancer, we avoided countries with small populations for the comparison. We selected the above four countries because their data in the WHO mortality database are thought to be complete [11]. We calculated average age-adjusted mortality rates, which included five 5-year periods from 1980–1984 through 2000–2004 and a 6-year period of 2005–2010 for these countries. Mortality rates for each year were calculated and joinpoint models were fitted for 1980–2010.

Databases used in this study are publicly available. Age-adjusted mortality rates were calculated using R version 3.1.0 (Institute for Statistics and Mathematics, Vienna, Austria; www.R-project.org) [12]. The joinpoint regression models were fitted using the Joinpoint software, version 4.0.0, from the Surveillance Research Program of the US National Cancer Institute (available at http://srab.cancer.gov/joinpoint) [13]. All calculated p values are two-sided, and all tests were conducted at a *p* = 0.05 level of statistical significance.

## Results

**Fig. 1:**
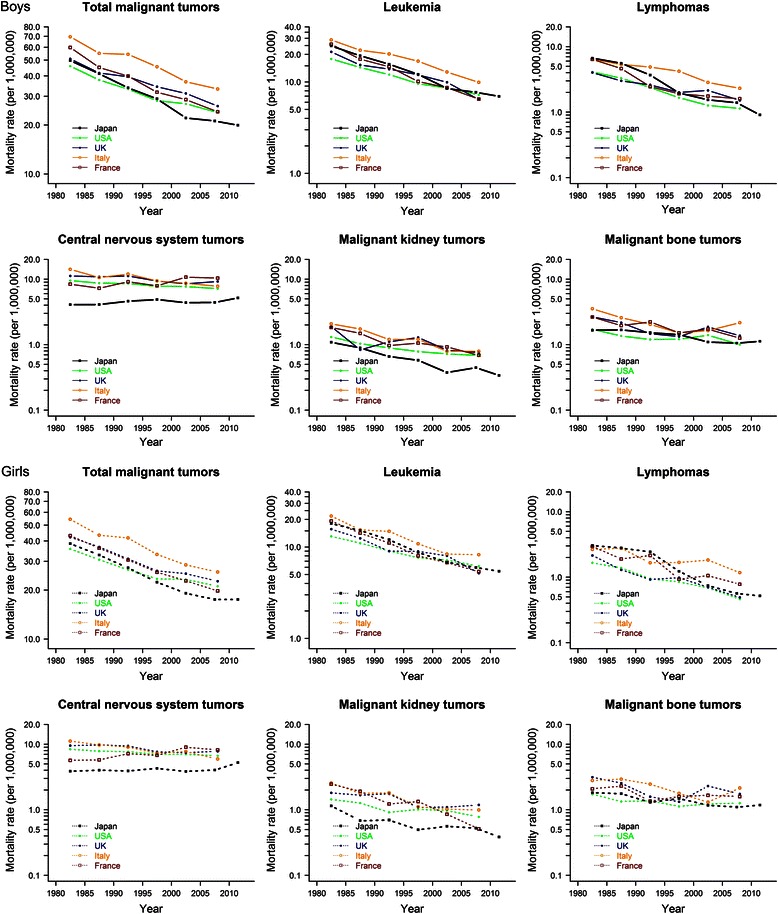
Childhood cancer mortality rate (per 1,000,000 population) in Japan and other selected countries (solid line, boys; dotted line, girls)

**Fig. 2:**
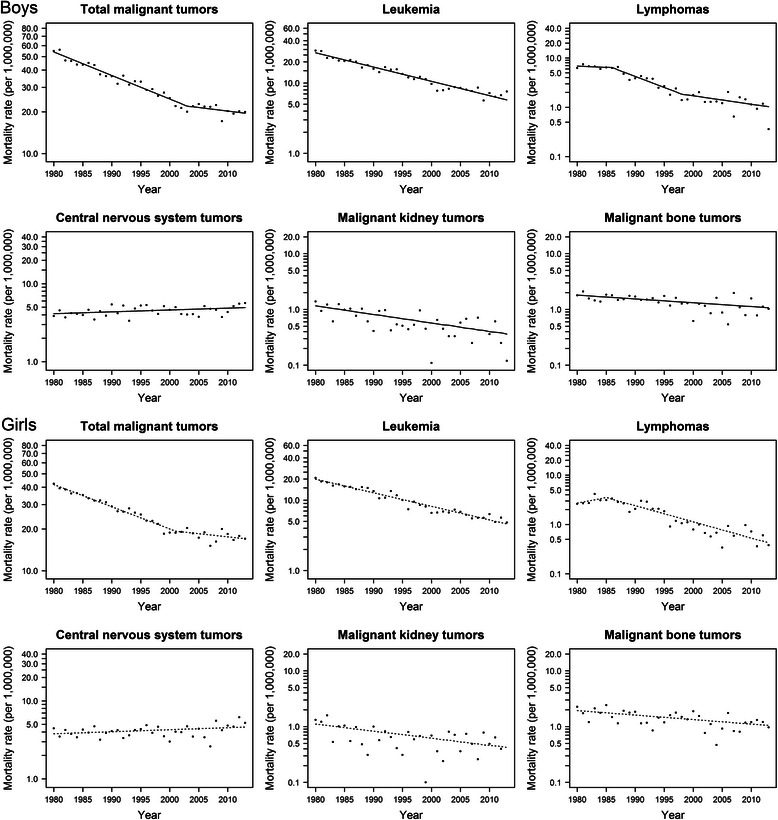
Age-standardized mortality rates and trends for childhood cancer per 1,000,000 population, Japan, 1980–2013 (solid line, joinpoint fitted trends for boys; dotted line, joinpoint fitted trends for girls; dots, observed rates)

A total of 20,924 childhood cancer deaths occurred in Japan during 1980–2013. Three hundred Japanese children died from cancer in 2013, of which leukemias were the most common diagnoses (33.7 %), followed by CNS tumors (29.3 %). Age-adjusted mortality rates are shown in Fig. 1. For all cancers combined, the mortality rate during 2010–2013 was 19.9 for boys and 17.5 for girls, per 1,000,000 population in Japan. Fig. 2 shows mortality rates of each year and trends after 1980 for all cancers combined and other site-based categories in Japan. The annual percent change for each joinpoint segment and AAPC are shown in Tables 1 and 2. For Japanese boys, mortality of all cancers combined decreased significantly (APC = −3.8, *p* <0.05) from 54.9 to 20.0 per 1,000,000 population from 1980 to 2003, followed by modest fluctuations after 2003 with a non-significant APC of −1.2 %. For Japanese girls, a similar mortality decline occurred from 1980 through 2001 (APC = −3.6, *p* <0.05), after which the rate was stable with a non-significant APC of–1.0 %.

**Table 1:** The APC and AAPC of childhood cancer mortality rates in boys

Country	Trend 1		Trend 2		Trend 3		Trend 4		AAPC	
	Years	APC	Years	APC	Years	APC	Years	APC	2000-2010	Last 5 Obs
Total malignant tumors										
Japan	1980-2003	−3.8^*^	2003-2013	−1.2					−2.0^*^	−1.2
USA	1980-1985	−4.1^*^	1985-1998	−3.0^*^	1998-2003	−0.2	2003-2010	−2.8^*^	−2.0^*^	−2.8^*^
United Kingdom	1980-2010	−2.4^*^							−2.4^*^	−2.4^*^
Italy	1980-1989	−4.8^*^	1989-1994	3.2	1994-1999	−7.5^*^	1999-2010	−2.0^*^	−2.0^*^	−2.0^*^
France	1980-1988	−5.1^*^	1988-2010	−3.0^*^					−3.0^*^	−3.0^*^
Leukemia										
Japan	1980-2013	−4.6^*^							−4.6^*^	−4.6^*^
USA	1980-2010	−3.6^*^							−3.6^*^	−3.6^*^
United Kingdom	1980-2010	−3.9^*^							−3.9^*^	−3.9^*^
Italy	1980-2010	−3.8^*^							−3.8^*^	−3.8^*^
France	1980-2010	−5.3^*^							−5.3^*^	−5.3^*^
Lymphomas										
Japan	1980-1986	−1.2	1986-1998	−9.8^*^	1998-2013	−3.9^*^			−3.9^*^	−3.9^*^
USA	1980-2010	−5.4^*^							−5.4^*^	−5.4^*^
United Kingdom	1980-2010	−3.4^*^							−3.4^*^	−3.4^*^
Italy	1980-2010	−3.7^*^							−3.7^*^	−3.7^*^
France	1980-2010	−5.8^*^							−5.8^*^	−5.8^*^
Central nervous system tumors										
Japan	1980-2013	0.5^*^							0.5^*^	0.5^*^
USA	1980-2010	−1.1^*^							−1.1^*^	−1.1^*^
United Kingdom	1980-2010	−1.0^*^							−1.0^*^	−1.0^*^
Italy	1980-2010	−2.1^*^							−2.1^*^	−2.1^*^
France	1980-2010	1.2^*^							1.2^*^	1.2^*^
Malignant kidney tumors										
Japan	1980-2013	−3.5^*^							−3.5^*^	−3.5^*^
USA	1980-2010	−2.5^*^							−2.5^*^	−2.5^*^
United Kingdom	1980-2010	−2.4^*^							−2.4^*^	−2.4^*^
Italy	1980-2010	−3.8^*^							−3.8^*^	−3.8^*^
France	1980-2010	−3.9^*^							−3.9^*^	−3.9^*^
Malignant bone tumors										
Japan	1980-2013	−1.6^*^							−1.6^*^	−1.6^*^
USA	1980-2010	−1.4^*^							−1.4^*^	−1.4^*^
United Kingdom	1980-2010	−2.3^*^							−2.3^*^	−2.3^*^
Italy	1980-2010	−2.5^*^							−2.5^*^	−2.5^*^
France	1980-2010	−2.3^*^							−2.3^*^	−2.3^*^

^*^p <0.05

APC = annual percent change; AAPC = average annual percent change; Obs = observations

**Table 2:** The APC and AAPC of childhood cancer mortality rates in girls

Country	Trend 1		Trend 2		Trend 3		Trend 4		AAPC	
	Years	APC	Years	APC	Years	APC	Years	APC	2000-2010	Last 5 Obs.
Total malignant tumors										
Japan	1980-2001	−3.6^*^	2001-2013	−1.0					−1.3^*^	−1.0
USA	1980-1995	−2.9^*^	1995-2010	−1.0^*^					−1.0^*^	−1.0^*^
United Kingdom	1980-1998	−3.2^*^	1998-2010	−1.0					−1.0	−1.0
Italy	1980-1989	−4.6^*^	1989-1994	1.4	1994-2003	−5.8^*^	2003-2010	1.6	−0.7	1.6
France	1980-2010	−3.0^*^							−3.0^*^	−3.0^*^
Leukemia										
Japan	1980-2013	−4.3^*^							−4.3^*^	−4.3^*^
USA	1980-1998	−3.5^*^	1998-2010	−1.7^*^					−1.7^*^	−1.7^*^
United Kingdom	1980-2010	−3.7^*^							−3.7^*^	−3.7^*^
Italy	1980-2010	−3.7^*^							−3.7^*^	−3.7^*^
France	1980-2010	−5.0^*^							−5.0^*^	−5.0^*^
Lymphomas										
Japan	1980-1985	5.2	1985-2013	−7.3^*^					−7.3^*^	−7.3^*^
USA	1980-2010	−4.5^*^							−4.5^*^	−4.5^*^
United Kingdom	1980-1985	−18.9^*^	1985-2010	−2.4^*^					−2.4^*^	−2.4^*^
Italy	1980-2010	−2.6^*^							−2.6^*^	−2.6^*^
France	1980-2010	−4.7^*^							−4.7^*^	−4.7^*^
Central nervous system tumors										
Japan	1980-2013	0.6^*^							0.6^*^	0.6^*^
USA	1980-2010	−0.9^*^							−0.9^*^	−0.9^*^
United Kingdom	1980-2010	−1.1^*^							−1.1^*^	−1.1^*^
Italy	1980-2010	−2.3^*^							−2.3^*^	−2.3^*^
France	1980-2010	1.6^*^							1.6^*^	1.6^*^
Malignant kidney tumors										
Japan	1980-2013	−2.9^*^							−2.9^*^	−2.9^*^
USA	1980-2010	−2.2^*^							−2.2^*^	−2.2^*^
United Kingdom	1980-2010	−1.8^*^							−1.8^*^	−1.8^*^
Italy	1980-2010	−4.0^*^							−4.0^*^	−4.0^*^
France	1980-2010	−4.8^*^							−4.8^*^	−4.8^*^
Malignant bone tumors										
Japan	1980-2013	−1.8^*^							−1.8^*^	−1.8^*^
USA	1980-1988	−5.3^*^	1988-2010	−0.1					−0.1	−0.1
United Kingdom	1980-1995	−5.4^*^	1995-2010	2.7					2.7	2.7
Italy	1980-2010	−2.0^*^							−2.0^*^	−2.0^*^
France	1980-2010	−1.1							−1.1	−1.1

*p <0.05

APC = annual percent change; AAPC = average annual percent change; Obs = observations

Compared with other countries, Japan showed the lowest mortality in all cancers combined for both sexes after 2000. Mortality from all childhood cancer declined during 2000–2010 with AAPCs between–2 % and–3 % in boys. For girls, declines were observed in all countries with AAPCs during 2000–2010 between–1 % and–3 %, although the changes in the UK and Italy were not statistically significant.

Rates for leukemia in Japan fell significantly over the whole study period with an APC of–4.6 % (*p* <0.05) in boys and–4.3 % in girls (*p* <0.05). Mortality from leukemia in the other countries showed a similar decline with AAPC between–2 % and–5 % for both boys and girls during 2000–2010.

Deaths from lymphomas among Japanese boys declined significantly during 1986–1998 (APC = −9.8) and 1998–2013 (APC = −3.9). For girls, mortality fell during 1985 to 2013 after a non-significant change between 1980 and 1985. The average annual change in 2000–2010 was–3.9 % (*p* <0.05) for boys and–7.3 % (*p* <0.05) for girls. Significant declines were also observed in the other countries after 2000.

The mortality rate for CNS tumors remained low in Japan during the whole period studied. In contrast with the dramatic decline in deaths from leukemia, mortality from CNS tumors increased from 1980 to 2013 by 0.5 % per year (*p* <0.05) for boys and 0.6 % per year (*p* <0.05) for girls. Declines were observed in the US, UK, and Italy, while in France the APC increased 1.2 % (*p* <0.05) for boys and 1.6 % (*p* <0.05) for girls since 1980.

Deaths from malignant kidney tumors and malignant bone tumors were rare in Japan, with mortality rates below two per 1,000,000 population for both sexes. Rates of kidney cancer declined in the study period by 3.5 % per year (*p* <0.05) for boys and 2.9 % per year (*p* <0.05) for girls. Declines were also found for malignant bone tumors, with APC of–1.6 % (*p* <0.05) for boys and–1.8 % (*p* <0.05) for girls. Similar declines occurred in Italy for both sexes. In the other three countries, mortality from malignant bone tumors fell in boys during the study period but remained stable in girls after 2000.

## Discussion

This updated analysis provides the latest information on trends in mortality from childhood cancer in Japan. We found that dramatic declines in mortality occurred during 1980–2003 for boys and 1980–2001 for girls. In the last 5 years, there have been modest but statistically insignificant reductions in mortality for both sexes.

The declines in childhood cancer mortality in the 1980s and 1990s are most likely due to improved survival. Early diagnosis and better therapies for childhood cancer over recent decades have greatly improved the prognosis of pediatric cancer patients [14]. The 5-year survival rate reached 79 % for children with cancer diagnosed between 1998 and 2000, based on a population-based report from Hiroshima, Japan [15]. The contribution of changes in cancer incidence to mortality decline remains unclear because of the absence of incidence data in Japan. A population-based study in Osaka, Japan that found a decline in the incidence rate of all cancers combined in the 1990s suggested that the constant decline in death from childhood cancer during 1973–2001 was primarily due to improved survival between the 1970s and 1980s and reduced incidence after the 1990s [16].

The impressive decrease in leukemia mortality is consistent with a substantial increase in survival from this disease, particularly in patients with acute lymphoblastic leukemia. Standardization of the applied protocol and the expanded use of chemotherapeutic agents and combination regimens have improved the treatment of childhood leukemia [17–19]. In the latest report on pediatric hematological malignancies from the Japan Society of Pediatric Hematology/Oncology, the 5-year overall survival rate for patients diagnosed between 2006 and 2010 reached 88.7 % for acute lymphoblastic leukemia and 75.2 % for acute myeloid leukemia [4].

Malignant CNS tumors continue to be the second largest contributor to cancer-related mortality in Japanese children. We found a slight but statistically significant increase in mortality of childhood CNS tumors in Japan. This increase in mortality implies a modest increase in incidence of CNS tumors. A recent study of mortality of CNS tumors by subtype between 1993 and 2013 indicates that mortality rises for the tumors of C71.7 (brain stem) and C71.5 (cerebral ventricle). Since patients with tumors in these sites have a poor prognosis, the trends of cancer incidence and mortality for these sites are thought to be similar. Therefore, the study author speculates that tumors occurring in these sites are increasing [20]. Although this explanation is reasonable, since childhood CNS tumors are rare and even rarer by subtype, whether there is a true increase in brain stem or cerebral ventricle tumors in Japan awaits confirmation. More importantly, our results highlight the necessity of establishing an active, nationwide, childhood cancer registry to provide reliable incidence information for Japan.

The lack of decline in the mortality rate of CNS tumors in recent years has also been reported in other countries. For example, a population-based study in Australia found a stable trend in mortality between 1998 and 2008. The author attributed this finding to fluctuations in CNS cancer incidence and stable survival rates [21].

Stable trends were observed in mortality of all cancers combined in recent years in Japan. Accompanying the rapid reduction in the number of deaths due to leukemia, the proportion of deaths from CNS tumors increased over the studied period. In 1980, leukemia accounted for nearly 52 % of childhood cancer deaths; by 2013, it accounted for 34 %. In contrast, CNS tumors’ share of all cancer deaths increased from about 9 % in 1980 to 29 % in 2013. With this recent shift in disease distribution, the number of deaths from leukemia has become similar to that from CNS tumors in Japan. This means that trends in mortality of CNS tumors tend to contribute more to the changes in mortality of all cancers combined. The recent statistically insignificant drop in mortality from all cancers combined is due in part to the rise in death from CNS tumors during the study period.

Our estimates of childhood cancer rates and trends for Japan are similar to those of four comparison countries, but some differences exist. For example, mortality from CNS tumors was lower in Japan than elsewhere. That difference might be explained by the relatively low disease incidence rate in Japan since treatment achievements from using the same regimens are similar in Japan and other developed countries. Study of the Osaka Cancer Registry showed an incidence of CNS tumors in children of about two per 100,000 population between 1988 and 2001 [16]. Even lower rates resulted from a study of childhood cancer incidence from 1993 to 2001 based on 15 population-based cancer registries in Japan: 1.5 per 100,000 population for boys and 1.4 for girls [22]. In the same period, a study from the Surveillance, Epidemiology, and End Results Program reported a higher childhood cancer incidence rate in the US, over three per 100,000 population [23].

Data consistency and accuracy should be considered when comparing Japan with other countries. Our study used the WHO mortality database, an established, international death file, to evaluate cancer trends. According to Mathers et al., for developed countries the quality of cause-of-death information is good and the coverage of populations is complete [11]. Hence, we consider our comparison between Japan and the other four countries in this study to be reasonable and reliable.

The statistical methods used to evaluate changes in mortality rates may have influenced the observed patterns. In this study, we described trend characteristics using joinpoint analysis, a method used extensively to describe mortality trends in epidemiological cancer studies. Joinpoint analysis allows identification of points of change and determination of the trends between joinpoints, considering variations within the relevant period. In addition, we calculated both APC and AAPC, which may provide complete characterization of the trend for the whole study period.

It is important to address the limitations when explaining and comparing results involving mortality statistics. First, as described above, the quality of data differs among countries in the WHO mortality database, and thus comparison is limited to those countries with good completeness and accuracy of death certification. Second, the rarity of childhood cancer deaths limits the analysis of changes in cancer mortality by subtype. Finally, no known study has measured the accuracy of cancer death certificate diagnoses for children with cancer. Although a study of all age groups reported 65 % agreement of cancer diagnoses between death certificates and hospital records, whether this is also true for pediatric cancer is unknown [24, 25].

## Conclusions

We provide an update on the recent trends of mortality of childhood cancer. The dramatic declines in the death rates of many childhood cancers, especially leukemia, represent treatment-related improvements in survival. Establishment of a nationwide, childhood cancer registry is required in Japan to improve surveillance of incidence and survival rates. Moreover, trends in cancer mortality should be monitored continuously.

## Abbreviations

WHO: World health organization
CNS: Central nervous system
APC: Annual percent change
ICD: International classification of diseases
AAPC: Average annual percent change
US or USA: United States
UK: United Kingdom
Obs: Observations
